# Assessment of metal(loid) and natural radionuclide pollution in surface sediments of an estuary affected by mining and phosphogypsum releases

**DOI:** 10.1007/s11356-024-34439-8

**Published:** 2024-08-08

**Authors:** José Luis Guerrero, Alejandro Barba-Lobo, Carmen Romero-Forte, Juan Pedro Bolívar

**Affiliations:** 1 Department of Integrated Sciences, Center for Natural Resources, Health and Environment (RENSMA), University of Huelva, Avda. Fuerzas Armadas s/n, 21071 Huelva, Spain; 2 Global Earth Change and Environmental Geology Research Group, Department of Biology and Geology, Physics and Inorganic Chemistry, Universidad Rey Juan Carlos, c/Tulipán s/n, 28933 Móstoles, Spain; 3 Department of Medical Radiation Sciences, Institute of Clinical Sciences, Sahlgrenska Academy at University of Gothenburg, 413 45 Gothenburg, Sweden

**Keywords:** Heavy metals, Natural radionuclides, Tinto River estuary, Acid mine drainage, Phosphogypsum

## Abstract

The prolonged impact over the Tinto River estuary by both the significant pollution by acid mine drainage (AMD) affecting this river and the polluted releases from phosphogypsum (PG) piles has led to the severe environmental degradation of this ecosystem. The aim of this work was to assess the current environmental quality of the Tinto River estuary through the study of the spatial distribution of metal(loid)s and natural radionuclides in the surface sediments from the channel edge. The sediments contain mean concentrations 5–20 times higher than the background values for pollutants such as Zn, As, Cu, Pb, or U, and up to two orders of magnitude higher for P. The studied sediments are heavily polluted by toxic heavy metals and metalloids (Pb, Zn, Cu, and As) according to the US EPA guidelines. Most of the analyzed sediment samples are also strongly polluted by long-lived natural radionuclides, mainly U-isotopes and ^210^Pb with concentrations up to one order of magnitude higher than unpolluted sediments, mostly due to the contribution by the PG leachates. The enrichment factors (EF) were extremely high (EF > 50) for As and very severe enrichment (25 ≤ EF < 50) for P, Cd, Zn, Cu, and Pb.

**Supplementary Information:**

The online version contains supplementary material available at 10.1007/s11356-024-34439-8.

## Introduction

**Fig. 1:**
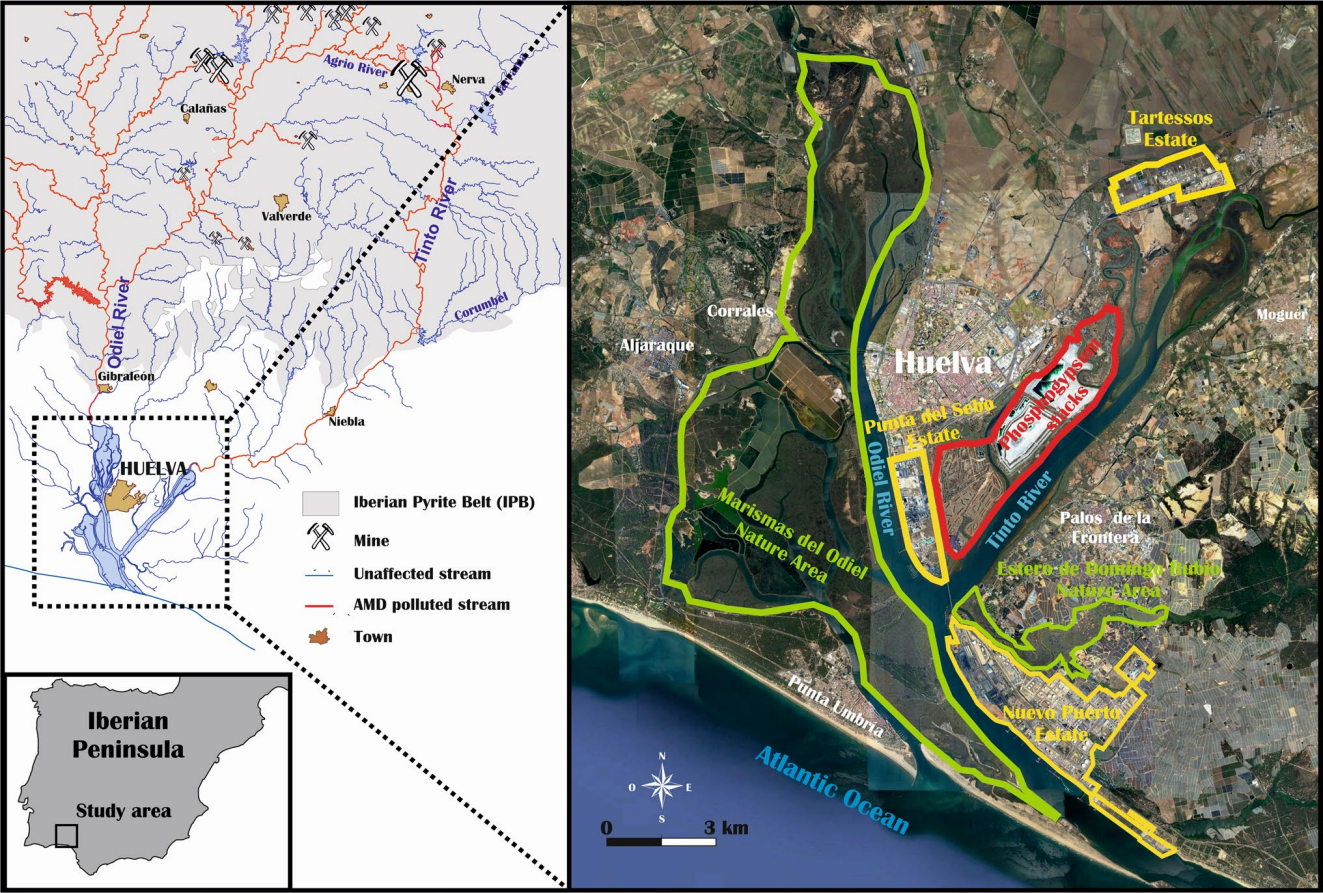
Map of the study area showing the main sites: industrial complex, mining areas, natural protected areas, and involved towns

The estuary of Huelva, called popularly “Ría de Huelva,” is formed by the common mouths of the Odiel and Tinto rivers, and represents a clear example of a chronically polluted area. One of the main concerns in this estuary is the degradation of soils, saltmarshes, and as consequence its habitats, including several protected areas of the Natura 2000 Network (Habitat Directive 1992), with a high biodiversity value and extremely vulnerable habitats. Their degradation is mainly due to the industrial releases that have been discharged since the 1960s, and the acid mine drainage (AMD) leachates coming from the IPB, where for the last two centuries intense mining activities have been developed (Fig. 1). The net transport of pollutants from the estuaries to the ocean is influenced by fluvial inputs and their geochemical behavior (Hierro et al. 2014). Estuaries act as a trap for metal(loid)s through sorption onto particulate matter, driven by solid-water interactions that result in net sedimentation (Bewers and Yeats 1989). Conversely, processes such as mineral dissolution, desorption, transformation, and the resuspension of bed sediments play a crucial role in the migration and fate of contaminants in aquatic environments, including their transport to the ocean (Zhou et al. 2003).

The IPB is one of the biggest metallogenic areas of massive sulfides in the world, cutting across the central and northern parts of Huelva Province. Mining activities in the IPB date back to the Third Millennium BC, although the large-scale exploitation of these deposits took place from the second half of nineteenth to twentieth century (Leblanc et al. 2000). The galleries, pits, and the stacking of tailings interact with waters and dissolved oxygen in them, generating AMD (Younger et al. 2002; Agboola et al. 2020), with pH = 2–4, and dissolved concentrations of SO_4_, Fe, heavy metals, metalloids, and natural radionuclides from both U- and Th-series up to four to five orders of magnitude higher than those found in unperturbed surface waters (Madejón et al. 2021; Guerrero et al. 2021a, 2023). Most of the AMD leachates generated in the IPB are collected by the Odiel and Tinto rivers catchments (around 3·10^3^ km^2^) (Nieto et al. 2013). When the waters of these rivers reach the estuary of Huelva, they are mixed with the seawaters, increasing suddenly their pH until neutral values, and therefore generating the precipitation of a high fraction of the contained chemical species depending on their chemical behavior (Hierro et al. 2014; Guerrero et al. 2021a).

On the other hand, in the estuary of Huelva a large industrial chemical complex was installed in the 1960s. This industrial complex generated a high environmental impact up to 2000 years, when the implementation of the “spill correction plan” finished, which was designed by consensus between the industrial complex and the government of Andalusia.

The five phosphoric acid production plants installed in Huelva worked for 45 years (1965 to 2010), and until 1998 they generated around 2.5 Mt/year of a waste called phosphogypsum (PG), which was stored in large piles on the salt marshes of the Tinto River, covering an area of about 1000 ha (Fig. 1), and releasing about 10 Mt/year of acid seawaters (pH = 1–2) used for the PG, containing heavy metals and natural radionuclides from U-series in levels about four to five orders of magnitude higher than not polluted surface waters (Bolívar et al. 2009; Más et al. 2006). From 1998 to 2010, the PG was pumped in a closed circuit, and the pumping waters were recirculated newly into the factories after the decantation of PG onto the PG stacks. PG is mainly composed by gypsum (CaSO_4_·2H_2_O) but contains high concentrations of ^238^U-series radionuclides, toxic metals, and other impurities such as remaining phosphoric acid (PG has a pH ≈ 2) and fluorine (Pérez-López et al. 2007; Bolívar et al. 2009). Therefore, PG is classified as a “naturally occurring radioactive material” (NORM) (EU 2013). In addition, the remaining phosphoric acid trapped in PG gives it a high acidity and polluting potential of the aqueous leachates produced by this waste (Pérez-Moreno et al. 2018).

**Fig. 2:**
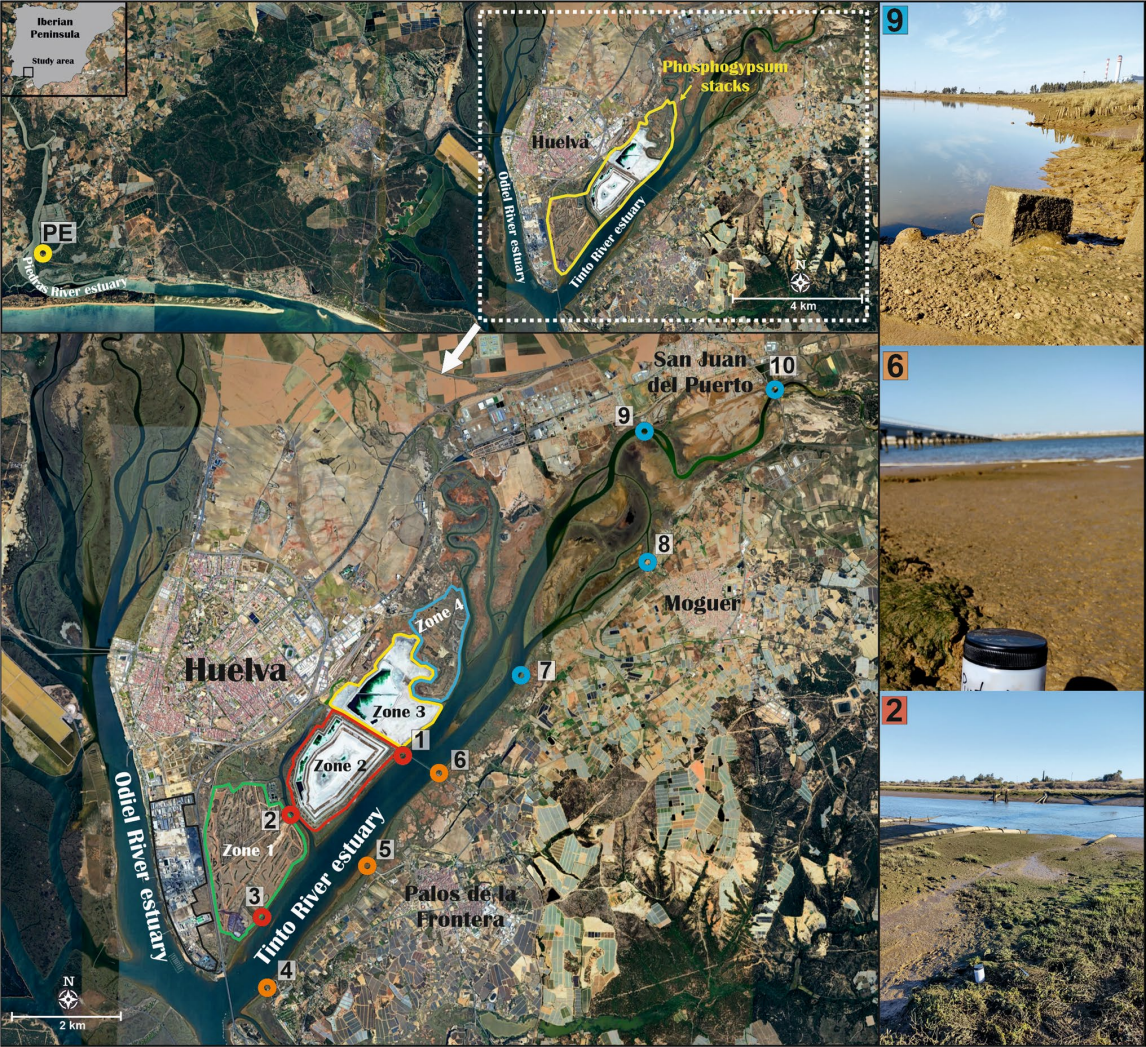
Location of the sampling points including the background from the Piedras River estuary (PE) and the samples from the Tinto River estuary (points 1 to 10). Right side: some pictures of the sampling locations

The PG repository, which stores around 100 Mt of PG, is often divided in four zones (Fig. 2). Zones 1 and 4 (around 550 ha in total), located to the north and south, respectively, are considered already restored. All restored and unrestored zones release “edge outflows,” formed by acidic polluted waters into the Tinto Estuary, with an average total flow of the releases around 10^5^ m^3^/year in the current state (Pérez-López et al. 2016).

These edge outflows show levels of ^238^U-series radionuclides, heavy metals and metalloids, acidity, and other chemical species such as NH_4_^+^, several orders of magnitude higher (two to five orders, depending on the pollutant) than unpolluted seawaters. Several studies have evaluated the behavior of the chemical species during the mixing of the outflow leachates with the estuarine waters, finding that some elements and heavy metals (e.g., Mn, Ni, Cd, As, Sb, and Co) were conservative, while other ones (e.g., Al, Fe, Cr, Zn, Cu, and Pb) precipitate and/or are adsorbed onto the particulate material (Guerrero et al. 2021b). The U-isotopes and ^210^Po showed a clear non-conservative behavior because they tend to be bound onto the formed precipitates, mainly composed by iron phosphate particles. All these facts demonstrate the potential high environmental impact that the PG stacks and the Tinto River waters can produce in the Tinto Estuary, and the urgency of taking effective restoration measures.

Only one study has been developed about the temporal evolution of the radioactive pollution at the estuary of Huelva from the year 2008, when started the close system of PG management (Villa et al. 2008). This study found that the decontamination rates for natural radionuclides (^226^Ra and ^210^Pb) from the sediments into the water followed an exponential reduction versus time and that natural cleaning of the bottom sediments by desorption is the most effective mechanism of decontamination. The corresponding half-lives were calculated by fittings to the experimental data for whole area of the estuary, finding about 6 and 3.5 years for ^226^Ra and ^210^Pb, respectively, but with very high uncertainties (Villa et al. 2009). 

Consequently, the releases coming from AMD and PG piles have become a global concern due to their high concentrations of different types of hazardous elements such as heavy metals, metalloids, and natural radionuclides, where AMD and PG releases can cause the pollution in the case of groundwater, surface water, and soil. Elevated concentrations of metal(loid)s significantly impact freshwater quality, marine ecosystems, and potentially human health. These effects occur through various pathways, including the consumption of contaminated seafood, freshwater fish, or exposure to metal(loid)-contaminated groundwater. Accordingly, the research conducted by Rosado et al. (2016) has raised concerns about adverse health effects associated with metal(loid) pollution in the Huelva Estuary. Notably, the Huelva province in Spain faces one of the highest mortality risks due to metal(loid) exposure (Benach et al. 2004). Therefore, the current situation poses risks to both human populations and ecosystems and has global significance for other polluted estuarine systems impacted by AMD and PG worldwide.

Considering the above facts, the main objective of this work was to assess the current environmental quality of the Tinto River estuary through the study of the spatial distribution of metal(loid)s and natural radionuclides in the surface sediments from the channel edge. This is very useful to find the correlations existing among the different types of hazardous elements and to identify the pollution sources that affect any sedimentary system such as AMD or PG releases that are of global interest. To the best of our knowledge, the spatial distribution of metal(loid)s and natural radionuclides along the Tinto River estuary has not been carried out for these last 20 years in any other previous works. This study shows the most updated pollution status of the Tinto River estuary for metal(loid)s and natural radionuclides, where the contamination levels were quantified and classified by using pollution indexes.

## Materials and methods

### Samplings and pretreatments

Surface sediment samples from 0 to 3 cm in depth were collected on October 25, 2022, along both banks of the Tinto Estuary (TE), from the intertidal zone of the channel. This small thickness was selected to assure that collected sediment was formed after 1998 year, when the new PG policy started by using a closed circuit, being the pumping PG waters returned into the phosphoric acid plants. This assumption is taken because previous works demonstrated that sedimentation rates in the Tinto Estuary are around 1 cm/year (San Miguel et al. 2004). Ten points were selected, three in the right bank next to PG piles (points 1, 2 and 3), other three points in the left bank (4, 5 and 6), and four points in the upper part of the estuary, very close to the zone of the estuary waters with the fluvial ones (points from 7 to 10) (see Fig. 2). Pictures of every one of these points and the coordinates of the samples, as well as a small description of them, are included in Fig. S1 and Table S1, respectively, of the Supplementary Material.

An aliquot of each sample without any pretreatment was reserved to granulometry tests, and the remaining amount was dried at 50 °C for avoiding dehydration of hydrated salts. An aliquot of the dried sample was milled (> 90% pass 70 μm) for the analysis of stable elements and natural radionuclides by different techniques and the determination of the mineralogical composition and another unmilled aliquot was preserved for the measurement of physicochemical parameters.

### Measurement techniques

The main physicochemical parameters (pH, electrical conductivity (EC), oxidation–reduction potential (ORP), and temperature (T)) of the sediment samples were measured in the laboratory with a Crison MM40 + portable multimeter by using an Ag/AgCl reference electrode (model 5048). The instruments were calibrated before using them, and the ORP was corrected to obtain the potential relative to the hydrogen electrode (Eh) according to Nordstrom and Wilde (1998). These parameters were determined by in the supernatant in a 1:2.5 (sample:distilled water) mix.

The granulometric analysis of the raw samples was carried out by mean of a laser diffraction particle size analyzer Malvern Mastersizer 2000 with a screen between 0.01 and 10,000 µm at the Central Research Services from the University of Huelva. Water was used as the dispersant liquid, and prior to the analysis the equipment was calibrated with different certified reference materials. Regarding the granulometric validation, the quality control was carried out by using three replicas for each sample, observing a relative difference < 10% for the great majority of the samples.

The mineralogical composition of the sediments was determined by X-ray diffraction (XRD), in combination with the Rietveld method, with a Bruker diffractometer (D8-Advance A25 with Cu radiation) and the associated software at the University of Seville Research, Technology and Innovation Centre (CITIUS). The analysis was of semiquantitative type, and each sample was well mixed with an internal standard (zincite (ZnO) in our case) to quantify the amorphous phase.

The measurement of major elements was carried out by using a Panalytical X-ray fluorescence (XRF) sequential spectrophotometer (AXIOS model) at the CITIUS. Minor and trace elements were analyzed by inductively coupled plasma optical emission spectrometry (ICP-OES) and inductively coupled plasma mass spectrometer (ICP-MS), using the equipment Varian 735 ES and Perkin Elmer Sciex ELAN 9000, respectively, at the Activation Laboratories (Actlabs) from Canada. The quality control (QC) was performed by the analysis at the beginning and end of each set of samples of Certified Standard Reference Materials. In addition, a duplicate for every 10 samples is run and internal control standards are analyzed.

The radioactive characterization of the samples was performed by applying both gamma-ray and alpha-particle spectrometry at the laboratories of the Radiation Physics and Environment Group (FRYMA) of the University of Huelva. For gamma-ray spectrometry, only a physical pretreatment consisting of drying to constant weight, homogenization, and compaction is required. Before measuring, it was necessary to use a previous efficiency calibration of the selected detectors, one of them was of range extended (XtRa) type (model GX3519, Canberra) and the other one was a well type (GCW3023, Canberra). The equipment and methodology are described in detail in Barba-Lobo et al. (2021a, b). For the measurement of the activity concentrations of alpha emitters, PIPS-type detectors were employed. The alpha-particle spectrometer (Alpha Analyst, Canberra) is composed of a silicon detector inside a vacuum chamber, whose counting efficiency is about 25%. Prior to the analysis, a sequential extraction technique was performed based on the use of tributyl phosphate (TBP) (Bolívar et al. 2000), subsequent electrodeposition onto stainless-steel disc (U–Th isotopes), microprecipitation for Ra, and self-deposition onto silver discs for the case of ^210^Po as describes in depth Pérez-Moreno et al. (2018). The QC for both radiometric techniques was conducted by participating in annual international proficiency tests (International Atomic Energy Agency [IAEA], Joint Research Center [JRC], and the Spanish Nuclear Safety Council [CSN]), and by analyzing both blank and Certified Standard Reference Materials, IAEA-326 (soil) and IAEA-434 (phosphogypsum), every batch of five samples.

### Data treatment

A principal component analysis (PCA) was performed facilitating the data interpretation by using the XLSTAT software (Lumivero 2023).

## Results and discussion

### Estuarine sediment background assessment

The first step to assess the environmental impact existing on a specific geographical area is to determine its reference geochemical baseline (background). For this, it is needed to find a non-impacted zone with similar geochemical substrate (Salminen and Gregorauskien 2000; Adriano 2001). The natural background level (NBL) can be defined as the concentration of a chemical or physical agent in the environment due to natural sources. In this context, NBL is useful to identify the current content of pollutants and to assess the degree of contamination by human activities. In Spain, soil protection policy was established with the publication of the Royal Decree, 9/2005 (Royal Decree, 9/2005), which is explained by Tarazona et al. (2005). Generic Reference Levels (GRLs) were settled in this Spanish Decree, which are the concentration of a contaminant in soil that does not result in a level of risk higher than the acceptable maximum for human health or ecosystems.

Due to the historical mining exploitation of the sulfide deposits located in the IPB, it is difficult to establish a background value for the Tinto Estuary. San Miguel et al. (2004) obtained a sedimentation rate on the surface sediments in this estuary around 1.2 cm/year. In previous studies (Guerrero et al. 2019, 2020), seven cores were collected on the right bank of the estuary (total depth: 13–16 m), just in the zones 2 and 3 of the stacks to study the impact of these deposits in the underlying sediments, concluding that the pollution due percolation of the leachates only reaches as maximum the first 50 cm below the contact. To establish a background of the Tinto Estuary, in this study the mean values of the concentrations of stable and radioactive elements of seven samples from these cores located from 3.6 to 12.1 m depth were used. These samples were chosen considering that the large-scale exploitation of the deposits began in the nineteenth century and the sedimentation rate of the estuary; therefore, it can be estimated that the sediments used were deposited between 400 and 1400 years ago, i.e., far before the increase of the pollution of this estuary due to modern exploitation.

Since the above commented problematic about the background of the Tinto Estuary, the authors decided to include in this study the background values of another location from the Huelva coast with similar characteristics. The estuary of the Piedras River was selected since previous works demonstrated that no mining activities has been developed on its basin, and, in addition, no releases are produced by the industries and/or towns located along its course (Fernández Caliani et al. 1997; Lario et al. 2016). In this case, the background values of this unimpacted area were calculated as the mean values of stable elements and natural radionuclide concentrations of three samples from the first 10-cm depth of a core collected in 2022 by the authors (Barba-Lobo et al. 2024) in the left bank of this estuary.

The background values for major and trace elements and natural radionuclides of both locations, Piedras and Tinto Estuary, can be consulted in Table S2 of the Supplementary Material. As can be checked the concentrations of most elements and natural radionuclides are very similar and, surprisingly, the concentrations of some metals such as Zn, As, Sn, Cu, or Pb are slightly higher in the Piedras Estuary, confirming that the values from the Tinto Estuary can be considered the natural background of this location.

### Physicochemical parameters

**Fig. 3:**
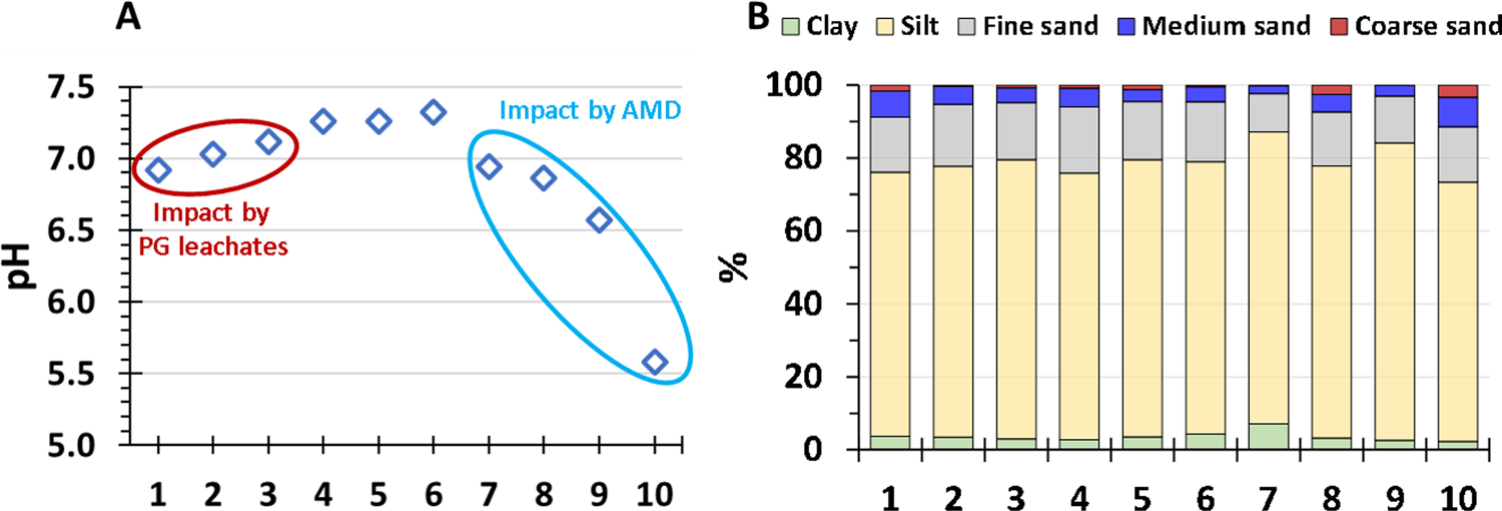
Values of pH (**A**) and granulometry (**B**) of the samples

The values of pH in the supernatant of the mix of sediments with distilled water are plotted in Fig. 3A. The values of EC and Eh can be consulted in Fig. S2 of the Supplementary Material. The pH values ranged from 5.6 to 7.3 and are in general influenced by the seawater (pH ≈ 8). The lower values were measured in the samples located in the upper part of the estuary due to the influence of the acid waters of the Tinto River polluted by AMD. It is also observed the impact of the acidic releases from the stacks in the sediments collected in the right bank, which showed slightly lower values than the located ones in the opposite bank of the estuary.

In Fig. 3B, the percentages of different grain size fractions are displayed according to the ISO 14688–1:2017: gravel (> 2 mm), coarse sand (630–2000 µm), medium sand (200–630 µm), fine sand (63–200 µm), silt (2–63 mm), and clay (< 2 µm). The particle size distribution curves of the samples can be consulted in Fig. S3 of the Supplementary Material. The granulometric characterization of the samples has an essential role since sediment grain size substantially influences the concentration of pollutants in marine sediments due to fine-grained sediments have a higher specific surface area favoring adsorption processes for heavy metals (Thuy et al. 2000) and radionuclides (Heldal et al. 2021). The sediments are composed in general by particles ranging from 1 to 1000 µm with peak in volume about 10 µm. Therefore, these sediments are mainly composed by silts (mean = 75%) and fine sand particles (mean = 15%). The clay fraction was lower than 5% in all samples except for sample 7 which reach a 7% of this finest fraction. Medium and coarse sands also showed small percentages with mean values of 5 and 1%, respectively. This analysis indicates that the sediments show a very similar granulometric distribution and can be classified as sandy silts according to the Shepard (1954) ternary diagram. The obtained results in this work are in accordance with previous studies developed in this estuary (López-González et al. 2006) and other ones from the Huelva coast such as the Piedras Estuary (Borrego et al. 1990) used as background in our study or the Guadiana Estuary (Delgado et al. 2012), indicating the homogeneity in the estuarine sediments from this coastal sector.

### Concentration of major, minor, and trace elements

**Fig. 4:**
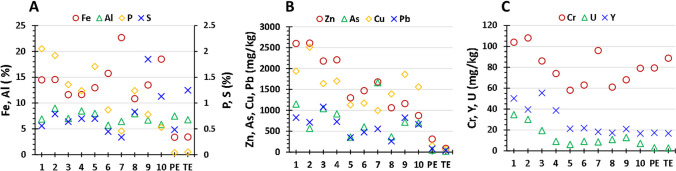
Concentration of some major (**A**) and minor and trace elements (**B**, **C**) in the sediment samples and background values. PE, Piedras Estuary; TE, Tinto Estuary

The concentrations of some major, minor, and trace elements of interest in the sediments and the backgrounds from the Piedras Estuary (PE) and Tinto Estuary (TE) are represented in Fig. 4. The concentrations of all the elements measured can be consulted in Tables S3 and S4 of the Supplementary Material. The metals with the highest concentrations in the sediment samples were Fe and Al. Fe showed a mean value of 15%, ranging from 11 to 23%. These concentrations are between 3- and sevenfold the background values, indicating a significant and uniform enrichment by Fe in the estuarine surface sediments, as can be expected due to the strong AMD pollution of the Tinto River. The highest concentrations were measured in the sediments located in the upper part of the estuary, probably due to the precipitation of Fe oxyhydroxysulfates (mainly schwertmannite) after the neutralization of the acidic waters from the Tinto River in the mixing zone (Carro et al. 2011; Hierro et al. 2014). Al had a mean value of 7% and a range from 6 to 9%. This metal tends to precipitate in the mixing zone mainly in the form of hydroxysulfates of Al such as basaluminite (Carro et al. 2011; Hierro et al. 2014). The concentrations were very similar to the background values (≈ 7%) and very uniform along the estuary, which indicates a low or no pollution by Al in the sediments.

The analysis of P concentration is very interesting since it is a very good tracer to assess the impact of the PG stacks due to its high mobility and concentration in the acidic releases from the stacks (Guerrero et al. 2019, 2021b). The concentration of P ranged from 0.5 to 2% (mean = 1.2%), and the highest values were measured, as can be expected, in the samples collected in the lower part of the estuary, closer to the stacks. These values are from one to two orders of magnitude higher than the background (≈ 0.05%), which demonstrate the strong pollution by P of the sediments from the Tinto Estuary due to the leachates from the stacks. S is also an interesting major element due to the high concentration of this elements in the leachates from the PG (≈ 1500 mg/L), and in the waters polluted by AMD of the Tinto River (≈ 1000 mg/L on its final stretch). The concentration of this element in the sediments showed a mean value of 0.80 mg/kg (range: 0.34–1.85 mg/kg). These values are in general similar to the background, which is consistent with the highly conservative behavior of this element after the neutralization of acidic leachates with high sulfur concentrations with seawater (Guerrero et al. 2021b).

Other pollutants such as Zn (mean = 1700 mg/kg), As (mean = 800 mg/kg), Cu (mean = 1600 mg/kg), Pb (mean = 650 mg/kg), or U (mean = 15 mg/kg) showed mean concentrations from 5 to 20 times higher than the background values, demonstrating the strong pollution of this estuarine environment. It is interesting to note how Zn, Y, and U showed notably higher concentrations in the sediments located in the bank next to the stacks, clearly indicating the influence of the edge outflows in the concentrations of these pollutants.

The concentrations of some pollutant metal(loid)s in the collected samples were assessed by comparing with the sediment quality guidelines (SQGs) proposed by the US EPA (US EPA 1977) and the NOAA (NOAA 1999). The US EPA establishes “non-polluted,” “moderately polluted,” and “heavily polluted” levels based on toxicity tests. These data were used to classify the sediments from Great Lakes harbors, and in the case of Pb and Zn were based on compilation of data from over 100 different harbors, while the ranges for the rest of pollutants are based of 260 samples from 34 harbors. The NOAA presents “effects range-low” (ERL) and “effects range-median” (ERM) values for estuarine and marine environments which represent the P10 and P50 percentiles of adverse biological effects (Table 1). These values were calculated for 9 trace metals, 13 individual polycyclic aromatic hydrocarbons (PAHs), 3 classes of PAHs, and 3 classes of chlorinated organic hydrocarbons based on the database assembled by Long et al. (1995). To provide quantitative information on how well the SQGs correctly predict toxicity in actual field conditions, an analysis was conducted (Long et al. 1998) with existing data compiled from many regional assessments conducted by NOAA and EPA. Matching chemistry and toxicity data from 1068 samples from the Atlantic, Gulf of Mexico, and Pacific coasts were compiled into a database. Several analyses were conducted with the data to investigate the predictive ability of the SQGs.

**Table 1:** Sediment quality guidelines (SQGs) proposed by the US EPA and NOAA (mg/kg dry weight)

Element	This study	USEPA			NOAA	
	Mean (range)	Non-polluted	Moderately polluted	Heavily polluted	ERL	ERM
Pb	647 (260–1080)	< 40	40–60	> 60	46.7	218
Zn	1714 (875–2610)	< 90	90–200	> 200	150	410
Cu	1590 (996–2510)	< 25	25–50	> 50	34	270
As	807 (359–1660)	< 3	3–8	> 8	8.2	70
Cd	3.0 (1.9–5.2)	-	-	> 6	1.2	9.6
Cr	80 (58–108)	< 25	25–75	> 75	81	370
Ni	29 (24–36)	< 20	20–50	> 50	20.9	51.6
Mn	306 (198–433)	< 300	300–500	> 500	-	-

*ERL*, effects range—low; *ERM*, effects range—median

The results showed that all the estuarine samples are heavily polluted by Pb, Zn, Cu, and As according to the US EPA guideline. The mean concentrations for the above elements in the sediments are far above the threshold of this degree of pollution, being up to two orders of magnitude higher for As. The concentrations of these elements are also higher than the ERM which represent concentrations above which adverse effects frequently occur. The studied samples are also from moderate to heavily polluted by Cr, moderately polluted by Ni, and most of the located ones near the PG stacks moderately polluted by Mn. Otherwise, these samples can be considered unpolluted by Cd. Previous studies as the one conducted by Rosado et al. (2016) assessed the bioavailability and toxicity of heavy metals in sediments from the Huelva Estuary, concluding that the sediments from the Huelva Estuary far exceed the threshold to be considered toxic, in the worst cases, by more than 50 times. In addition, Vicente-Martorell et al. (2009) analyzed the heavy metal concentrations in two species of fish from the Huelva Estuary and found high Cu and Zn levels in liver tissue of both species, in agreement with the higher total content and more bioavailability of these metals in water and sediments obtained in this study.

### Mineralogy

**Fig. 5:**
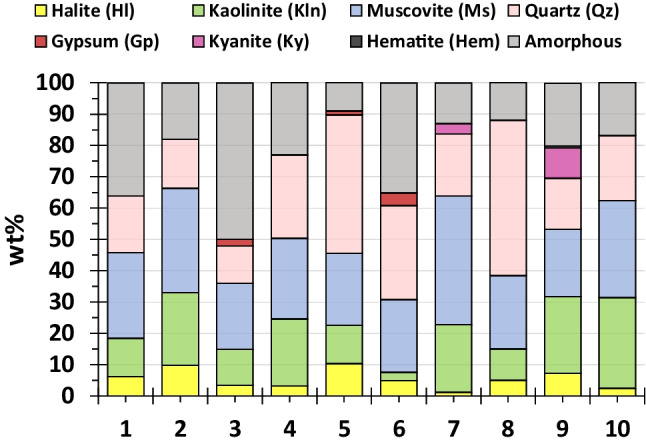
Percentage of crystalline mineral phases in the sediment samples

The mineralogical composition of the samples is displayed in Fig. 5. To note the high percentage of amorphous phase in the sediments, with a mean value of 22 wt% (range: 9–50 wt%). Regarding the crystalline phase, the sediments are mainly composed by silicates, as can be expected, with a percentage higher than 70 wt% in most cases. The main silicate minerals are quartz (range: 12–50 wt%), and aluminosilicates such as muscovite (range: 21–41 wt%) and kaolinite (range: 3–29 wt%), while kyanite was only found in the samples 7 (3 wt%) and 9 (10 wt%). These mineral phases agree with the observed ones by previous studies in this estuary such as Fernández Caliani et al. (1997) or Prudêncio et al. (2022). The presence of halite (range: 1 to 10 wt%) is due to the precipitation from the salty estuarine water retained in the sediment. It is remarkable the existence of gypsum in samples 3 (2.2 wt%), 5 (1.3 wt%), and 6 (4 wt%). The existence of gypsum in these sediments located in the surroundings of the PG stacks seems to indicate direct pollution of gypsum particles (PG is more than 95 wt% composed of gypsum) from these piles. There are two processes than can produce the dragging of these particles from the stacks into the estuarine sediments: 1) the surface runoff over the uncovered stacks and 2) moments of high spring tide and/or high flow of the Tinto River when the waters can reach to the border of the stacks. Hematite was only observed in the sample 9 with a value below 1 wt%. The low percentage of crystalline phases with iron indicates that this major metal is in the amorphous phase.

### Natural radionuclides

**Fig. 6:**
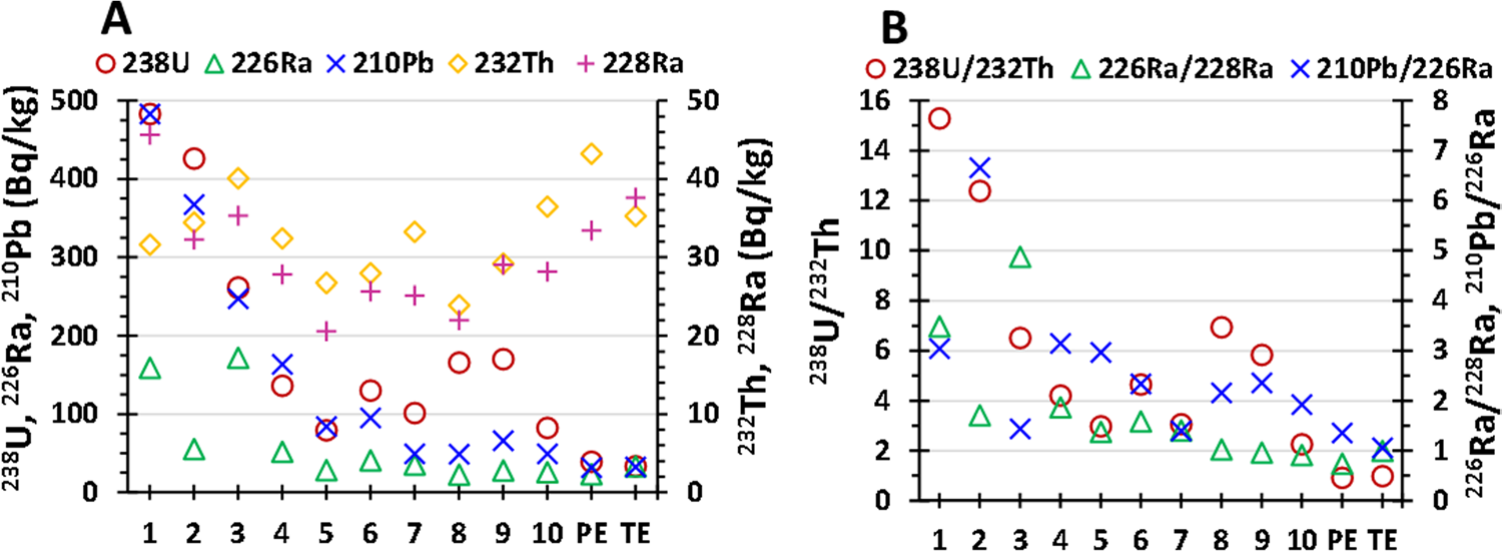
Activity concentration of natural radionuclides in the estuarine sediment samples and background values. *PE*, Piedras Estuary; *TE*, Tinto Estuary

There are two main pollution sources of natural radionuclides in the Tinto Estuary, the AMD polluting the Tinto River and the leachates from the PG stacks. In this work, the main radionuclides with long half-lives from the ^238^U- and ^232^Th-series, ^137^Cs and ^40^ K were studied. The concentrations of some of the studied radionuclides in the sediment samples and in the backgrounds (Piedras and Tinto estuaries) are displayed in Fig. 6A. In this case, the ^238^U-series radionuclide content of the sediments is highly relevant due to the high concentrations of natural radionuclides from this series in the leachates from the PG stacks, being in the order: ^238,234^U (10^2^ Bq/L), ^210^Po (10 Bq/L), and ^226^Ra and ^230^Th (1 Bq/L) (Gázquez et al. 2014; Pérez-Moreno et al. 2018). These values are from four to five orders of magnitude higher than the observed ones in unperturbed surface freshwater and seawater (De Vos and Tarvainen 2006; IAEA 2017), which demonstrates the extreme polluting potential of these acidic leachates. In addition, some activity ratios were calculated to deepen in the pollution processes by radionuclides affecting the sediments from the estuary (Fig. 6B). The concentration of all the studied natural radionuclides and the calculated activity ratios can be consulted in Tables S5 and S6, respectively, from the Supplementary Material.

The activity concentration of ^238^U-series radionuclides was in general notably higher in the sediments located in the bank next to the stacks (Fig. 6A). The mean activity concentration of ^238^U in the samples was around 200 Bq/kg, while the sediments located in the bank next to the stacks showed significantly higher activity concentrations, with a maximum close to 500 Bq/kg for the sample 1 clearly due to the edge outflows. The ^234^U showed secular equilibrium with ^238^U (^234^U/^238^U activity ratios ≈ 1) in the sediments and therefore a similar behavior in the estuary (Table S6). ^210^Pb had similar concentrations than the ^238^U in the samples located in the lower part of the estuary (samples 1 to 6), while in the upper part (samples 7 to 10) were lower. These lower concentrations of ^210^Pb in relation to the ^238^U concentrations in the upper part of the estuary are probably due to its lower mobility (Wei et al. 2011; Guerrero et al. 2020), and the higher influence by AMD since U contained in Tinto waters should precipitate at the beginning of the estuary where pH increases from 2–3 up to 5–7 (Hierro et al. 2013). ^226^Ra showed abnormally higher activity concentrations in the samples 1 and 3, above 150 Bq/kg, and below 60 Bq/kg in the rest of the samples. The lower concentration of ^226^Ra in relation to the ^238^U one is clearly related to the lower concentration of this radionuclide in the PG leachates as was previously indicated (two orders of magnitude lower).

The obtained results demonstrate that the sediments located closer to the stacks have a higher pollution by natural radionuclides from the ^238^U-series, and these concentrations decrease with the distance to the piles. The worldwide median concentration for ^238^U in unperturbed sediments is 35 Bq/kg (range: 16–110 Bq/kg), and for ^226^Ra is also 35 Bq/kg (range: 17–60 Bq/kg) (UNSCEAR 2000), in agreement with the obtained values in our reference backgrounds. Therefore, it seems clear that most of the sediments analyzed are strongly polluted by ^238^U and ^210^Pb, with values up to one order of magnitude higher than unpolluted sediments, while only two of the collected ones in the bank of the stacks are significantly polluted by ^226^Ra.

It is very interesting to compare the results obtained in this study with the obtained ones in a previous study conducted by Hierro et al. (2012), where surface samples were also collected in the Tinto Estuary between 2007 and 2009, before the cease of the phosphoric acid production in 2010. The results showed a mean ^238^U activity concentration of 540 Bq/kg in the estuarine sediments (range: 30–2500 Bq/kg) while ^226^Ra presented a mean activity concentration of 30 Bq/kg (range: 14 to 54 Bq/kg). Therefore, a decrease of around 60% of the mean concentration for ^238^U was observed in the estuarine sediments during the last decade, while ^226^Ra showed similar values except for the samples 1 and 3, probably due to the location of these ones closer to the stacks than the collected in this previous study. The decrease in the ^238^U concentrations in the last 10 years is probably related to two facts: on the one hand, the building of a new perimeter channel in 2014 in the zone 3 of the stacks, and the evaporation process developed in the acidic waters existing inside the zones 2 and 3 from the stacks to proceed with its restoration, with the consequence of decreasing the volume of polluted aqueous releases leachates into the Tinto Estuary.

The activity concentrations of ^232^Th in the samples ranged from 24 to 40 Bq/kg (mean = 32 Bq/kg), while for ^228^Ra ranged from 21 to 46 Bq/kg (mean = 29 Bq/kg). The concentrations of these radionuclides are relatively uniform along the estuary and similar to the worldwide median concentration in natural sediments which is 30 Bq/kg (range: 11–64 Bq/kg) (UNSCEAR 2000), and in the local backgrounds considered in this study. The above facts demonstrate that the inputs of ^232^Th-series radionuclides by the PG leachates and/or the AMD pollution of the Tinto River are negligible, on the one hand due to the low concentration of ^232^Th in both pollution sources and also the low mobility of this radioelement (Pérez-Moreno et al. 2018; Guerrero et al. 2021a). Therefore, these findings indicate that the concentrations of ^232^Th-series radionuclides throughout the study area are similar to unimpacted estuarine sediments. The results agree with the obtained ones by Hierro et al. (2012), where ^232^Th concentrations ranged from 15 to 41 Bq/kg.

In addition, the activity ratios between radionuclides from the ^238^U-series and ^232^Th-series are a helpful tool to determine the degree of relative radioactive pollution in the sediments due to the releases from the PG stacks. In this regard, the ^238^U/^232^Th and ^226^Ra/^228^Ra activity ratios were studied. Unpolluted sediments should present activity ratios around 1 as was observed in the reference backgrounds (Fig. 6A), while higher ratios indicate a secondary source of ^238^U-series radionuclides in the sediments, i.e., pollution due to PG leachates. The ^238^U/^232^Th activity ratio showed a mean value of 6.4 in the collected sediments, ranging from 2.3 (sample 10) and 15.3 (sample 1). The higher activity ratios were observed in the sediments collected in the right bank of the Tinto Estuary as can be expected. Hierro et al. (2012) obtained a mean value of 19 (range: 4–100) for this ratio, notably higher than the mean value of this study ratifying the decrease in the pollution by ^238^U in the sediments from the Tinto Estuary in the last 10 years. The ^226^Ra/^228^Ra activity ratio presented values from 0.91 (sample 10) to 4.9 (sample 3), with a mean of 1.9. This ratio showed significantly lower values than the ^238^U/^232^Th ratio, and in the samples 8, 9, and 10 were around 1 (unpolluted sediments). These observations ratify that the pollution by ^226^Ra mainly reaches the sediments located next to the stacks and the strong and uniform pollution by ^238^U in the sediments from the Tinto Estuary due to its high mobility. The ^210^Pb/^226^Ra activity ratio showed a mean value of 2.7 (range: 1.4–6.6). These values ratify the stronger pollution by ^210^Pb than by ^226^Ra in the surface sediments of the estuary.

The mean concentration of ^137^Cs, in surface sediments was 1.95 Bq/kg (range: 0.83–3.03 Bq/kg). The atmospheric deposition of ^137^Cs took place during in the 1950s and 1960s due to atmospheric nuclear weapon tests worldwide, justifying the low activity concentration of this artificial radionuclide in the surface sediments. In a previous study conducted by Bolivar et al. (1995), an average value around 7.0 Bq/kg (range: 1.9–23.2 Bq/kg) of ^137^Cs in the surface sediments from the Tinto Estuary was obtained, which agrees with the lower value obtained in the current study. These concentrations are very similar to the obtained ones in other coastal surface sediments from unpolluted locations worldwide (Pan et al. 2011, Uddin and Behbehani 2018; Zhang et al. 2019). Finally, the ^40^ K activity concentrations showed a uniform distribution in the estuarine sediments with a mean value of 440 Bq/kg (range: 344–483 Bq/kg). These values are similar to the background ones for natural soils (mean: 400 Bq/kg, range: 140–850 Bq/kg) established by the UNSCEAR (2000).

### Enrichment factors (EF)

The enrichment factor (EF) for the main pollutants (including stable elements and natural radionuclides) was calculated to evaluate the degree of anthropogenic contamination in the sediments. The EF were calculated as follows:

1 $$EF=\frac{{({~}^{[X]}\!\left/ \!{~}_{[Al]}\right.)}_{\mathrm{sample}}}{{({~}^{[X]}\!\left/ \!{~}_{[Al]}\right.)}_{\mathrm{background}}}$$

where [*X*]_sample_ is the concentration of the element or radionuclide in the sample, [Al]_sample_ is the concentration of Al in the sample, [*X*]_background_ is the background value of an element or radionuclide, and [Al]_background_ is the concentration of Al in the background. Al was chosen as normalizing element due to two facts: firstly, this element represents the quantity of aluminosilicates, which is the predominant carrier phase for metal sediments in coastal sediments and its natural concentration tends to be uniform (Alexander et al. 1993), and, secondly, according to the hypothesis that metal concentrations vary consistently with the concentration of aluminum, unless metals are of anthropogenic origin (Summers et al. 1996). Because of the above, Al have been widely used as reference element for geochemical normalization, e.g., Soto-Jiménez and Páez-Osuna (2001), Selvaggi et al. (2019), or Sousa et al. (2019), including natural radionuclides (Blanco-Rodríguez et al. 2014).

An EF value of 1 indicates a natural sediment origin for the element, while values above 1 point out enrichment by natural processes (biota input) or anthropic pollution (Zhang and Liu 2002). EF values lower than 1 can reflect mobilization and loss of the element in relation to Al or point out an overestimation of the background metal contents (Mil-Homens et al. 2006). The degree of enrichment was interpreted based on Birch and Davies (2003): EF < 1 “no enrichment,” 1 ≤ EF < 3 “minor enrichment,” 3 ≤ EF < 5 “moderate enrichment,” 5 ≤ EF < 10 “moderately severe enrichment,” 10 ≤ EF < 25 “severe enrichment,, 25 ≤ EF < 50 “very severe enrichment,” and EF > 50 “extremely severe enrichment.”

**Fig. 7:**
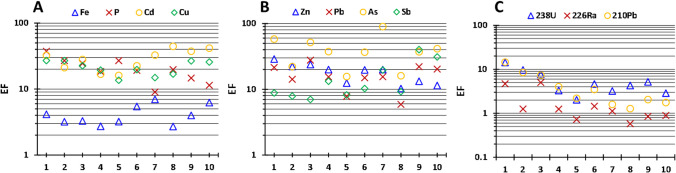
Enrichment factors (EF) of the main pollutants in the samples

The EF of some of the main stable pollutants and natural radionuclides are plotted in Fig. 7. All the calculated EF can be consulted in Table S7 of the Supplementary Material. The pollutants with EF above 1 in the sediments, indicating an anthropogenic source, and the ranges obtained are as follows: Fe (2.7–7.0), P (9–37), Cd (16–44), Zn (10–29), As (16–90), Sn (1.8–11), Sb (7.0–40), Cu (14–27), Pb (6.0–28), Y (0.88–3.2), ^238^U (2.0–14), ^226^Ra (0.58–5.0), and ^210^Pb (1.3–14.6). According to these values, in the worst cases, extremely enrichment was reached for As (samples 1, 3, and 7), very severe enrichment for P (samples 1, 2, and 5), Cd (samples 1, 3, 7, 8, 9, and 10), Zn (sample 1), Sb (samples 9 and 10), Cu (samples 1, 2, 9, and 10) and Pb (sample 3), and severe enrichment for Sn (sample 9), ^238^U (sample 1), and ^210^Pb (sample 1). The EF values show a clear spatial distribution for P and ^238^U-series radionuclides, with higher values in the lower part of the estuary due to the input of these elements into the sediments by the releases from the PG stacks. These findings demonstrate again the strong pollution of the sediments from the Tinto Estuary nowadays.

### Principal component analysis (PCA)

**Fig. 8:**
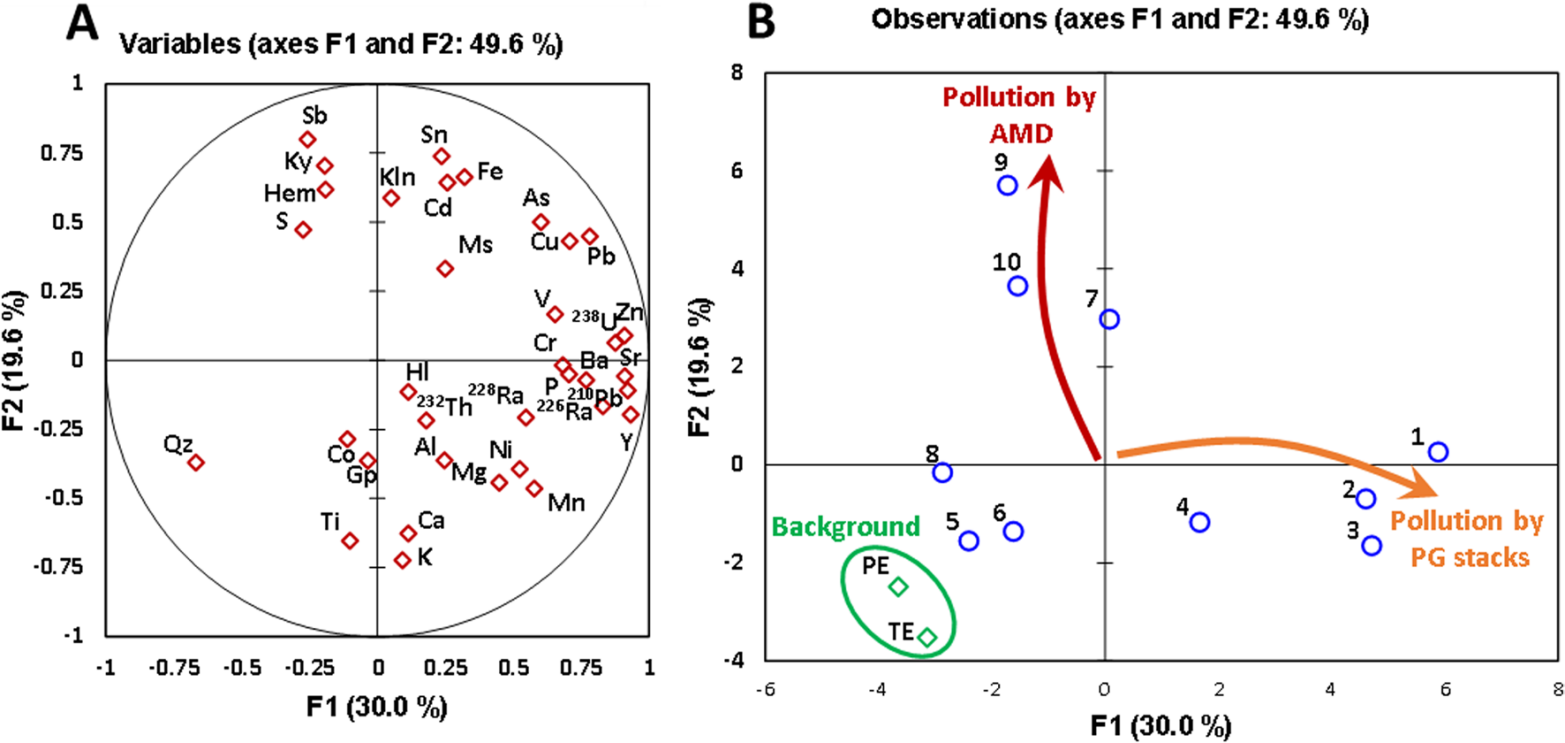
PCA results. **A** Loading plot of variables and **B** score plot of observations. *PE*, Piedras Estuary; *TE*, Tinto Estuary

To deepen the understanding of the pollution sources affecting the Tinto River Estuary, a principal component analysis (PCA) was performed. In this analysis, all the estuarine samples and the background values for the Piedras and Tinto estuaries were included. Of these samples, the concentration of elements, the activity concentration of the main ^238^U- and ^232^Th-series radionuclides, and the mineralogical composition were used in the analysis to know their relationships and improve the knowledge about the pollution processes that affects the saltmarsh sediments. The first two factors explain the 49.6% (F1: 30.0% and F2: 19.6%) of the data set total variance as can be observed in the plots from Fig. 8. This percentage is relatively low, probably due to the complexity of the pollution sources and large number of pollutants affecting this estuarine environment. The contribution of F3 and F4 is 15 and 10%, respectively, and the plots of F1 vs. F3 and F4 can be consulted in Figs. S4 and S5 of the Supplementary Material.

The F1 factor shows high positive scores for P, Zn, Sr, Ba, Cu, Pb, Y, and the ^238^U-series radionuclides (Fig. 8A). Phosphorus and the ^238^U-series radionuclides can be considered the main tracers of the pollution by the PG stacks due to their high concentrations in the edge outflows and mobility (Guerrero et al. 2019; 2020). The high scores of metals such as Zn, Cu, or Pb which could also be influenced by the AMD pollution seem to indicate a high release of these ones by the outflows from the PG stacks. In the negative side of F1, the mineral quartz, which is not related to any pollution source, stands out. In the PCA for observations (Fig. 8B), the samples with the highest positive scores are the ones located in the bank next the PG stack (samples from 1 to 3). The sample 4 is also located in the positive part of the F1 axis but with a low score, while the remaining sediment samples located in the opposite bank to the PG piles and in the upper part of the estuary are in the negative side of this axis. The samples with the highest negative score for F1 are the background from Piedras and Tinto estuaries. According to the above, the positive F1 component is related to the pollution by the acidic releases from the PG stacks.

Regarding the F2 factor is positively contributed by Fe, Cd, Sb, Sn, As, Cu, or Pb. These metal(loid)s are clearly related to the pollution by AMD on the estuary. The minerals hematite (iron oxide) and kyanite (aluminosilicate), which seem to be related to mining pollution due to the precipitation of Fe and Al, respectively, also contribute positively to this factor. The samples with the highest positive scores are 9 and 10, which were collected in the upper zone of the estuary, where pollution by AMD due the influence of the fluvial waters is higher. On the contrary, in the negative side of F2, the background samples clearly stand out. All of this indicates that positive F2 factor is closely related to the pollution of the sediments by AMD. Otherwise, elements such as K, Ti, or Ca, located in the negative side of F2 do not seem to be influenced by neither AMD nor PG leachates.

Finally, it is remarkable the location of the backgrounds (PE and TE) in the same corner of Fig. 8B and far from the studied samples, which ratify the similarity between them and the no pollution of these samples by the studied pollution sources.

## Conclusions

The current environmental quality of the Tinto River estuary (around 2022 year), which is affected by both mining and phosphogypsum leachates, was assessed through the study of the spatial distribution of metal(loid)s and natural radionuclides in surface sediments from the channel edge. The main conclusions obtained were as follows:

1. The sediments from the Tinto Estuary have a uniform granulometric distribution, being mainly composed by silts and fine sand particles. These sediments show a high proportion of amorphous phase (mean ≈ 20%) and are mainly composed of quartz and aluminosilicates such as muscovite and kaolinite.
2. The concentrations of P in the sediments are from one to two orders of magnitude higher than the background, which demonstrates the strong enrichment by this element of the sediments from the Tinto Estuary due to the leachates from the phosphogypsum stacks. Other pollutants such as Zn, As, Cu, Pb, or U showed mean concentrations from 5 to 20 times higher than the background values, demonstrating the strong pollution of this estuarine environment. Zn, Y, and U presented notably higher concentrations in the sediments located in the bank next to the stacks, clearly indicating the influence of the edge outflows on their concentrations.
3. All the estuarine samples are heavily polluted by toxic heavy metals and metalloids such as Pb, Zn, Cu, and As, according to the US EPA guideline. The concentrations of these elements are also higher than the “effects range-median” (ERM) established by the NOAA.
4. Most of the sediment samples analyzed are strongly polluted by natural radionuclides, mainly U-isotopes and ^210^Pb with concentrations up to one order of magnitude higher than unpolluted sediments, while only some sediments located next to piles are significantly polluted by ^226^Ra. The higher activity concentrations were observed in the sediments collected in the bank next to the stacks due to the leachates released from the PG stacks. A decrease around 60% in the mean concentration for ^238^U was observed in the estuarine sediments in the last decade.
5. The enrichment factor (EF) values indicate anthropic pollution (EF > 1) in the estuarine sediments for Fe, P, Cd, Zn, As, Sn, Sb, Cu, Pb, Y, ^238^U, ^226^Ra, and ^210^Pb. According to these values, in the worst cases, extremely enrichment for As; very severe enrichment for P, Cd, Zn, Cu, and Pb; and severe enrichment for Sn, ^238^U, and ^210^Pb were reached.
6. The PCA helps to discriminate the source of the pollutants. P, Zn, Sr, Ba, Cu, Pb, Y, and the ^238^U-series radionuclides seem to be highly influenced by the acidic releases from the PG stacks, while Fe, Cd, Sb, Sn, As, and Cu are mainly related to the AMD pollution.

## Supplementary Information

Below is the link to the electronic supplementary material.

Supplementary file1 (DOCX 2193 KB)

## Data Availability

All data and materials as well as software application or custom code support their published claims and comply with field standards.
