# Supplementary material for: Assessment of metal(loid) and natural radionuclide pollution in surface sediments of an estuary affected by mining and phosphogypsum releases
Source: Environ Sci Pollut Res Int. 2024 Aug 8;31(39):51489–503. doi: 10.1007/s11356-024-34439-8 (PMC11374832; doi:10.1007/s11356-024-34439-8)
Supplement: Supplementary file 1 — Supplementary file1 (DOCX 2193 KB) [file 11356_2024_34439_MOESM1_ESM.docx]

**Supplementary Material**

| **Point** | **UTM (WGS84)** | **Description** |
| --- | --- | --- |
| 1 | 29S 686120, 4124649 | Right bank of the Tinto estuary, close to the right upper corner of the zone 2 of the PG stacks |
| 2 | 29S 684095, 4123501 | Estero del Rincón, between the zones 1 and 2 of the PG stacks. |
| 3 | 29S 683596, 4121577 | Right bank of the Tinto estuary, at the zone 1 of the PG stacks and close to the roasted pyrite ashes pile. |
| 4 | 29S 683745, 4120300 | Muelle de la Reina, left bank of the Tinto estuary. |
| 5 | 29S 685527, 4122587 | Muelle de la Calzadilla, left bank of the Tinto estuary |
| 6 | 29S 686813, 4124358 | Left bank of the Tinto estuary, opposite to the point 1. |
| 7 | 29S 688309, 4126155 | Puerto de Santa, Moguer. |
| 8 | 29S 690614, 4128372 | Muelle de la Ribera, Moguer. |
| 9 | 29S 690552, 4130865 | Old pier from San Juan del Puerto. |
| 10 | 29S 692917, 4131577 | A-491 road over the Tinto River at San Juan del Puerto. |

Table S1. Coordinates and brief description of the sampling locations.

| Area | Al  (%) | Ca  (%) | Fe  (%) | K  (%) | Mg  (%) | Na  (%) | P  (%) | S  (%) | Ti  (%) | Cd  (mg/kg) |
| --- | --- | --- | --- | --- | --- | --- | --- | --- | --- | --- |
| PE | 7.48 | 0.26 | 3.41 | 1.81 | 0.80 | 1.42 | 0.041 | 0.49 | 0.53 | 0.30 |
| TE | 6.79 | 2.31 | 3.43 | 2.09 | 1.07 | 1.49 | 0.054 | 1.25 | 0.45 | 0.10 |
| Area | V  (mg/kg) | Cr  (mg/kg) | Mn  (mg/kg) | Ni  (mg/kg) | Co  (mg/kg) | Zn  (mg/kg) | As  (mg/kg) | Sr  (mg/kg) | Sn  (mg/kg) | Sb  (mg/kg) |
| PE | 101 | 79 | 275 | 34 | 79 | 310 | 46 | 85 | 17.7 | 1.2 |
| TE | 98 | 89 | 324 | 31 | 12 | 89 | 20 | 142 | 2.9 | 1.6 |
| Area | Ba  (mg/kg) | Cu  (mg/kg) | Pb  (mg/kg) | Y  (mg/kg) | Th  (mg/kg) | U  (mg/kg) | ^238^U  (Bq/kg) | ^234^U  (Bq/kg) | ^226^Ra  (Bq/kg) | ^210^Pb  (Bq/kg) |
| PE | 314 | 154 | 79.1 | 17.2 | 10.7 | 3.0 | 38.9 ±2.6 | 38.7±1.9 | 23.2±1.1 | 31.4±2.7 |
| TE | 272 | 70 | 37.8 | 16.7 | 8.7 | 2.6 | 33.1±1.7 | 34.1±1.7 | 33.3±1.7 | 32.5±1.6 |
| Area | ^232^Th  (Bq/kg) | ^228^Ra  (Bq/kg) | ^228^Th  (Bq/kg) | ^40^K  (Bq/kg) | ^238^U/^232^Th | ^226^Ra/^228^Ra | ^226^Ra/^234^U | ^210^Pb/^226^Ra | ^234^U/^238^U |  |
| PE | 43.2±2.2 | 31.7±1.9 | 33.4±1.4 | 636±27 | 0.91± 0.08 | 0.74±0.06 | 0.60±0.04 | 1.35±0.13 | 1.00±0.8 |  |
| PE | 35.3±1.8 | 37.6±1.9 | 33.1±1.7 | 709±35 | 0.95±0.07 | 0.89±0.06 | 0.98±0.07 | 1.01± 0.07 | 1.03±0.07 |  |

Table S2. Background values of stable elements and natural radionuclides. PE: Piedras estuary. TE: Tinto Estuary.

| Sample | Al | Ca | Cl | Fe | K | Mg | Na | P | S | Si | Ti | O | LOI |
| --- | --- | --- | --- | --- | --- | --- | --- | --- | --- | --- | --- | --- | --- |
| 1 | 6.92 | 1.22 | 2.55 | 14.5 | 1.57 | 1.11 | 1.57 | 2.05 | 0.56 | 12.5 | 0.28 | 32.6 | 21.3 |
| 2 | 9.01 | 1.09 | 4.31 | 14.6 | 2.10 | 1.75 | 2.11 | 1.92 | 0.79 | 16.4 | 0.37 | 40.0 | 4.2 |
| 3 | 7.02 | 1.23 | 3.40 | 11.6 | 1.87 | 1.31 | 1.85 | 1.36 | 0.64 | 15.2 | 0.46 | 34.3 | 18.6 |
| 4 | 8.49 | 1.18 | 2.77 | 11.7 | 1.94 | 1.43 | 1.51 | 1.24 | 0.70 | 17.8 | 0.47 | 38.4 | 11.3 |
| 5 | 8.02 | 0.97 | 3.84 | 13.0 | 1.87 | 1.56 | 1.88 | 1.71 | 0.70 | 14.6 | 0.33 | 35.6 | 14.7 |
| 6 | 5.73 | 2.12 | 3.59 | 15.8 | 1.63 | 1.22 | 1.88 | 0.87 | 0.45 | 14.5 | 0.33 | 33.5 | 16.3 |
| 7 | 6.45 | 0.41 | 1.17 | 22.7 | 1.60 | 0.82 | 0.96 | 0.46 | 0.34 | 14.4 | 0.35 | 34.8 | 14.6 |
| 8 | 7.95 | 0.73 | 3.11 | 10.9 | 1.75 | 1.09 | 1.57 | 1.24 | 0.83 | 16.8 | 0.34 | 36.1 | 16.8 |
| 9 | 6.72 | 0.4 | 3.38 | 13.5 | 1.54 | 0.97 | 1.52 | 0.78 | 1.85 | 12.1 | 0.28 | 31.4 | 24.8 |
| 10 | 5.87 | 0.25 | 2.78 | 18.5 | 1.58 | 0.71 | 1.30 | 0.53 | 1.13 | 12.3 | 0.32 | 31.2 | 22.8 |
| Min. | 5.73 | 0.25 | 1.17 | 10.9 | 1.54 | 0.71 | 0.96 | 0.46 | 0.34 | 12.1 | 0.28 | 31.2 | 4.2 |
| Mean | 7.22 | 0.96 | 3.09 | 14.7 | 1.75 | 1.20 | 1.62 | 1.22 | 0.80 | 14.6 | 0.35 | 34.8 | 16.5 |
| Median | 6.97 | 1.03 | 3.245 | 14.0 | 1.69 | 1.17 | 1.57 | 1.24 | 0.70 | 14.5 | 0.34 | 34.5 | 16.6 |
| Max. | 9.01 | 2.12 | 4.31 | 22.7 | 2.1 | 1.75 | 2.11 | 2.05 | 1.85 | 17.8 | 0.47 | 40.0 | 24.8 |
| SDM | 0.35 | 0.17 | 0.27 | 1.14 | 0.06 | 0.10 | 0.11 | 0.18 | 0.14 | 0.62 | 0.02 | 0.90 | 1.9 |

Table S3. Concentration of major elements (%) in the samples. LOI: Loss on ignition. SDM: standard deviation of the mean.

| Sample | Cd | V | Cr | Mn | Ni | Co | Zn | As | Sr | Sn | Sb | Ba | Cu | Pb | Y | Th | U |
| --- | --- | --- | --- | --- | --- | --- | --- | --- | --- | --- | --- | --- | --- | --- | --- | --- | --- |
| 1 | 3.3 | 112 | 104 | 321 | 31.6 | 23.3 | 2600 | 1150 | 494 | 15 | 14 | 478 | 1940 | 826 | 50.2 | 7.8 | 34.6 |
| 2 | 2.8 | 111 | 108 | 308 | 35.7 | 28.3 | 2610 | 569 | 338 | 19 | 16.3 | 449 | 2510 | 711 | 39.6 | 8.5 | 30.1 |
| 3 | 2.9 | 109 | 86 | 433 | 32.2 | 25.2 | 2180 | 1040 | 377 | 18 | 11.3 | 631 | 1640 | 1080 | 55.4 | 9.9 | 19.4 |
| 4 | 2.1 | 92 | 74 | 405 | 35.3 | 26.9 | 2210 | 913 | 272 | 19 | 25.8 | 477 | 1700 | 728 | 38.6 | 8 | 9.1 |
| 5 | 1.9 | 69 | 58 | 276 | 24 | 15.8 | 1300 | 359 | 183 | 6 | 15.1 | 289 | 1130 | 348 | 21.1 | 6.6 | 6.1 |
| 6 | 1.9 | 76 | 63 | 320 | 23.9 | 17.5 | 1470 | 602 | 208 | 9 | 13.5 | 357 | 1170 | 477 | 21.8 | 6.9 | 9.1 |
| 7 | 3.1 | 119 | 96 | 293 | 30.2 | 18.6 | 1680 | 1660 | 148 | 20 | 29.1 | 425 | 996 | 556 | 18.2 | 8.2 | 8.5 |
| 8 | 5.2 | 73 | 61 | 232 | 27.5 | 19.8 | 1060 | 366 | 166 | 9 | 16.8 | 279 | 1390 | 260 | 17.2 | 5.9 | 10.9 |
| 9 | 3.7 | 92 | 68 | 269 | 25.9 | 20.3 | 1160 | 715 | 230 | 30 | 61.9 | 201 | 1860 | 823 | 20.8 | 7.2 | 12.7 |
| 10 | 3.6 | 93 | 79 | 198 | 24.6 | 14.3 | 874 | 696 | 177 | 24 | 41.8 | 490 | 1560 | 662 | 16.6 | 9 | 7.1 |
| Min | 1.9 | 69 | 58 | 198 | 23.9 | 14.3 | 874 | 359 | 148 | 6 | 11.3 | 201 | 996 | 260 | 16.6 | 5.9 | 6.1 |
| Mean | 3.1 | 95 | 79.7 | 306 | 29.1 | 21.0 | 1714 | 807 | 259 | 17 | 24.6 | 408 | 1590 | 647 | 30.0 | 7.8 | 14.8 |
| Median | 3.0 | 93 | 76.5 | 301 | 28.9 | 20.1 | 1575 | 706 | 219 | 19 | 16.55 | 437 | 1600 | 687 | 21.5 | 7.9 | 10 |
| Max. | 5.2 | 119 | 108 | 433 | 35.7 | 28.3 | 2610 | 1660 | 494 | 30 | 61.9 | 631 | 2510 | 1080 | 55.4 | 9.9 | 34.6 |
| SDM | 0.32 | 5.6 | 5.74 | 22.6 | 1.43 | 1.50 | 203 | 126 | 35 | 2.3 | 5.10 | 40.05 | 143 | 77 | 4.63 | 0.38 | 3.2 |

Table S4. Concentration of minor and trace elements (mg/kg) in the samples. SDM: standard deviation of the mean.

| Sample | ^238^U | ^234^U | ^226^Ra | ^210^Pb | ^232^Th | ^228^Ra | ^228^Th | ^40^K | ^137^Cs |
| --- | --- | --- | --- | --- | --- | --- | --- | --- | --- |
| 1 | 483 ± 43 | 507 ± 45 | 159 ± 7 | 483 ± 20 | 31.6 ± 1.6 | 45.7 ± 2.9 | 39.9 ± 1.7 | 465 ± 22 | 2.28 ± 0.48 |
| 2 | 426 ± 14 | 438 ± 14 | 55 ± 2 | 367 ± 11 | 34.4 ± 1.7 | 32.3 ± 2.4 | 35.3± 1.2 | 469 ± 15 | 2.78 ± 0.50 |
| 3 | 261 ± 9 | 254 ± 9 | 172 ± 20 | 247 ± 9 | 40.1 ± 2.0 | 35.3 ± 2.6 | 37.5 ± 1.2 | 474 ± 15 | 3.03 ± 0.52 |
| 4 | 136 ± 5 | 143 ± 6 | 52 ± 2 | 163 ± 8 | 32.4 ± 1.6 | 27.8 ± 2.5 | 31.5 ± 1.2 | 405 ± 15 | 1.89 ± 0.49 |
| 5 | 79 ± 4 | 83 ± 4 | 28 ± 1 | 84 ± 6 | 26.7 ± 1.3 | 20.6 ± 1.7 | 24.9 ± 0.9 | 394 ± 12 | 0.83 ± 0.37 |
| 6 | 130 ± 9 | 116 ± 9 | 41 ± 1 | 95 ± 7 | 27.9 ± 1.4 | 25.7 ± 2.3 | 28.0 ± 1.0 | 414 ± 14 | 1.67 ± 0.47 |
| 7 | 101 ± 4 | 105 ± 4 | 35 ± 2 | 49 ± 4 | 33.2 ± 1.7 | 25.2 ± 2.0 | 31.7 ± 1.4 | 483 ± 22 | 1.11 ± 0.35 |
| 8 | 166 ± 4 | 176 ± 5 | 22 ± 1 | 48 ± 3 | 23.9 ± 1.2 | 22.0 ± 1.6 | 23.2 ± 1.0 | 344 ± 16 | 1.74 ± 0.34 |
| 9 | 170 ± 5 | 177 ± 5 | 28 ± 1 | 66 ± 5 | 29.2 ± 1.5 | 29.0 ± 2.4 | 41.1 ± 1.8 | 469 ± 23 | 1.58 ± 0.44 |
| 10 | 82 ± 3 | 92 ± 4 | 26 ± 1 | 49 ± 4 | 36.5 ± 1.8 | 28.1 ± 2.2 | 40.8 ± 1.8 | 467 ± 22 | 2.58 ± 0.45 |
| Min | 79 | 83 | 22 | 48 | 24 | 20.6 | 23 | 344 | 0.83 |
| Mean | 203 | 209 | 62 | 165 | 32 | 29.2 | 33 | 438 | 1.95 |
| Median | 151 | 159 | 38 | 90 | 32 | 28.0 | 34 | 466 | 1.82 |
| Max. | 483 | 507 | 172 | 483 | 40 | 45.7 | 41 | 483 | 3.03 |
| SDM | 45 | 47 | 18 | 48 | 1.5 | 2.3 | 2.1 | 15 | 0.23 |

Table S5. Activity concentration of natural radionuclides (Bq/kg) in the estuarine sediment samples. SDM: standard deviation of the mean.

| Sample | ^238^U/^232^Th | ^226^Ra/^228^Ra | ^226^Ra/^234^U | ^210^Pb/^226^Ra | ^234^U/^238^U |
| --- | --- | --- | --- | --- | --- |
| 1 | 15.3 ± 1.5 | 3.48 ± 0.27 | 0.31 ± 0.03 | 3.04 ± 0.18 | 1.05 ± 0.13 |
| 2 | 12.4 ± 0.7 | 1.71 ± 0.14 | 0.126 ± 0.006 | 6.65 ± 0.30 | 1.03 ± 0.05 |
| 3 | 6.51 ± 0.39 | 4.87 ± 0.66 | 0.68 ± 0.08 | 1.44 ± 0.17 | 0.97 ± 0.05 |
| 4 | 4.20 ± 0.27 | 1.87 ± 0.18 | 0.363 ± 0.019 | 3.15 ± 0.19 | 1.05 ± 0.06 |
| 5 | 2.96 ± 0.22 | 1.38 ± 0.13 | 0.343 ± 0.023 | 2.97 ± 0.23 | 1.04 ± 0.08 |
| 6 | 4.64 ± 0.40 | 1.58 ± 0.15 | 0.350 ± 0.029 | 2.33 ± 0.20 | 0.90 ± 0.09 |
| 7 | 3.05 ± 0.19 | 1.40 ± 0.14 | 0.337 ± 0.023 | 1.39 ± 0.15 | 1.03 ± 0.05 |
| 8 | 6.94 ± 0.39 | 1.02 ± 0.09 | 0.128 ± 0.007 | 2.16 ± 0.18 | 1.06 ± 0.04 |
| 9 | 5.83 ± 0.33 | 0.96 ± 0.09 | 0.158 ± 0.009 | 2.36 ± 0.22 | 1.04 ± 0.04 |
| 10 | 2.26 ± 0.14 | 0.91 ± 0.09 | 0.278 ± 0.018 | 1.92 ± 0.20 | 1.12 ± 0.06 |
| Min | 2.3 | 0.91 | 0.13 | 1.39 | 0.90 |
| Mean | 6.4 | 1.92 | 0.31 | 2.74 | 1.03 |
| Median | 5.2 | 1.49 | 0.33 | 2.35 | 1.04 |
| Max. | 15.3 | 4.87 | 0.68 | 6.65 | 1.12 |
| SDM | 1.3 | 0.40 | 0.05 | 0.48 | 0.02 |

Table S6. Activity ratio of natural radionuclides in the estuarine sediment samples. SDM: standard deviation of the mean.

| Samples | Fe | P | Ti | Cd | V | Cr | Mn | Ni | Co | Zn | As |
| --- | --- | --- | --- | --- | --- | --- | --- | --- | --- | --- | --- |
| 1 | 4.1 | 37.4 | 0.61 | 32.4 | 1.12 | 1.15 | 0.97 | 0.99 | 2.0 | 29 | 58 |
| 2 | 3.2 | 26.9 | 0.62 | 21.1 | 0.85 | 0.92 | 0.72 | 0.86 | 1.8 | 22 | 22 |
| 3 | 3.3 | 24.5 | 0.99 | 28.0 | 1.08 | 0.94 | 1.29 | 1.00 | 2.1 | 24 | 52 |
| 4 | 2.7 | 18.4 | 0.84 | 16.8 | 0.75 | 0.67 | 1.00 | 0.90 | 1.9 | 20 | 37 |
| 5 | 3.2 | 26.9 | 0.62 | 16.1 | 0.60 | 0.55 | 0.72 | 0.65 | 1.2 | 12 | 16 |
| 6 | 5.4 | 19.2 | 0.87 | 22.5 | 0.92 | 0.84 | 1.17 | 0.91 | 1.8 | 20 | 37 |
| 7 | 7.0 | 9.0 | 0.82 | 32.6 | 1.28 | 1.14 | 0.95 | 1.02 | 1.7 | 20 | 90 |
| 8 | 2.7 | 19.7 | 0.65 | 44.4 | 0.64 | 0.59 | 0.61 | 0.75 | 1.5 | 10 | 16 |
| 9 | 4.0 | 14.7 | 0.63 | 37.4 | 0.95 | 0.78 | 0.84 | 0.84 | 1.8 | 13 | 37 |
| 10 | 6.2 | 11.4 | 0.82 | 41.6 | 1.10 | 1.03 | 0.71 | 0.91 | 1.4 | 11 | 41 |
| Min. | 2.7 | 9.0 | 0.61 | 16.1 | 0.6 | 0.6 | 0.6 | 0.7 | 1.2 | 10.2 | 15.6 |
| Mean | 4.2 | 20.8 | 0.75 | 29.3 | 0.9 | 0.9 | 0.9 | 0.9 | 1.7 | 18.2 | 40.5 |
| Median | 3.6 | 19.4 | 0.73 | 30.2 | 0.9 | 0.9 | 0.9 | 0.9 | 1.8 | 19.8 | 37.2 |
| Max. | 7.0 | 37.4 | 0.99 | 44.4 | 1.3 | 1.2 | 1.3 | 1.0 | 2.1 | 28.8 | 89.6 |
| SDM | 0.5 | 2.7 | 0.04 | 3.2 | 0.1 | 0.1 | 0.1 | 0.0 | 0.1 | 1.9 | 7.0 |
| Samples | Sn | Sb | Cu | Pb | Y | ^238^U | ^226^Ra | ^210^Pb | ^232^Th | ^228^Th |  |
| 1 | 5.1 | 8.8 | 27 | 21 | 2.95 | 14.3 | 4.7 | 14.6 | 0.88 | 1.19 |  |
| 2 | 5.0 | 7.9 | 27 | 14 | 1.79 | 9.7 | 1.3 | 8.5 | 0.74 | 0.65 |  |
| 3 | 6.1 | 7.0 | 23 | 28 | 3.21 | 7.6 | 5.0 | 7.4 | 1.10 | 0.91 |  |
| 4 | 5.3 | 13.2 | 19 | 15 | 1.85 | 3.3 | 1.2 | 4.0 | 0.73 | 0.59 |  |
| 5 | 1.8 | 8.2 | 14 | 8 | 1.07 | 2.0 | 0.72 | 2.2 | 0.64 | 0.46 |  |
| 6 | 3.7 | 10.2 | 20 | 15 | 1.55 | 4.6 | 1.4 | 3.5 | 0.94 | 0.81 |  |
| 7 | 7.4 | 19.6 | 15 | 15 | 1.15 | 3.2 | 1.1 | 1.6 | 0.99 | 0.70 |  |
| 8 | 2.7 | 9.2 | 17 | 5.9 | 0.88 | 4.3 | 0.6 | 1.3 | 0.58 | 0.50 |  |
| 9 | 10.6 | 40.0 | 27 | 22 | 1.26 | 5.2 | 0.8 | 2.0 | 0.84 | 0.78 |  |
| 10 | 9.7 | 30.9 | 25.7 | 20.25 | 1.15 | 2.87 | 0.89 | 1.75 | 1.20 | 0.86 |  |
| Min. | 1.8 | 7.0 | 13.6 | 5.87 | 0.88 | 2.03 | 0.58 | 1.27 | 0.58 | 0.46 |  |
| Mean | 5.7 | 15.5 | 21.3 | 16.49 | 1.68 | 5.71 | 1.78 | 4.68 | 0.86 | 0.75 |  |
| Median | 5.2 | 9.7 | 21.1 | 15.44 | 1.40 | 4.46 | 1.18 | 2.83 | 0.86 | 0.74 |  |
| Max. | 10.6 | 40 | 27.1 | 27.62 | 3.21 | 14.30 | 5.00 | 14.58 | 1.20 | 1.19 |  |
| SDM | 0.89 | 3.6 | 1.6 | 2.08 | 0.25 | 1.20 | 0.52 | 1.35 | 0.06 | 0.07 |  |

Table S7. Enrichment Factors (EF) of pollutants in the sediment samples. SDM: standard deviation of the mean.


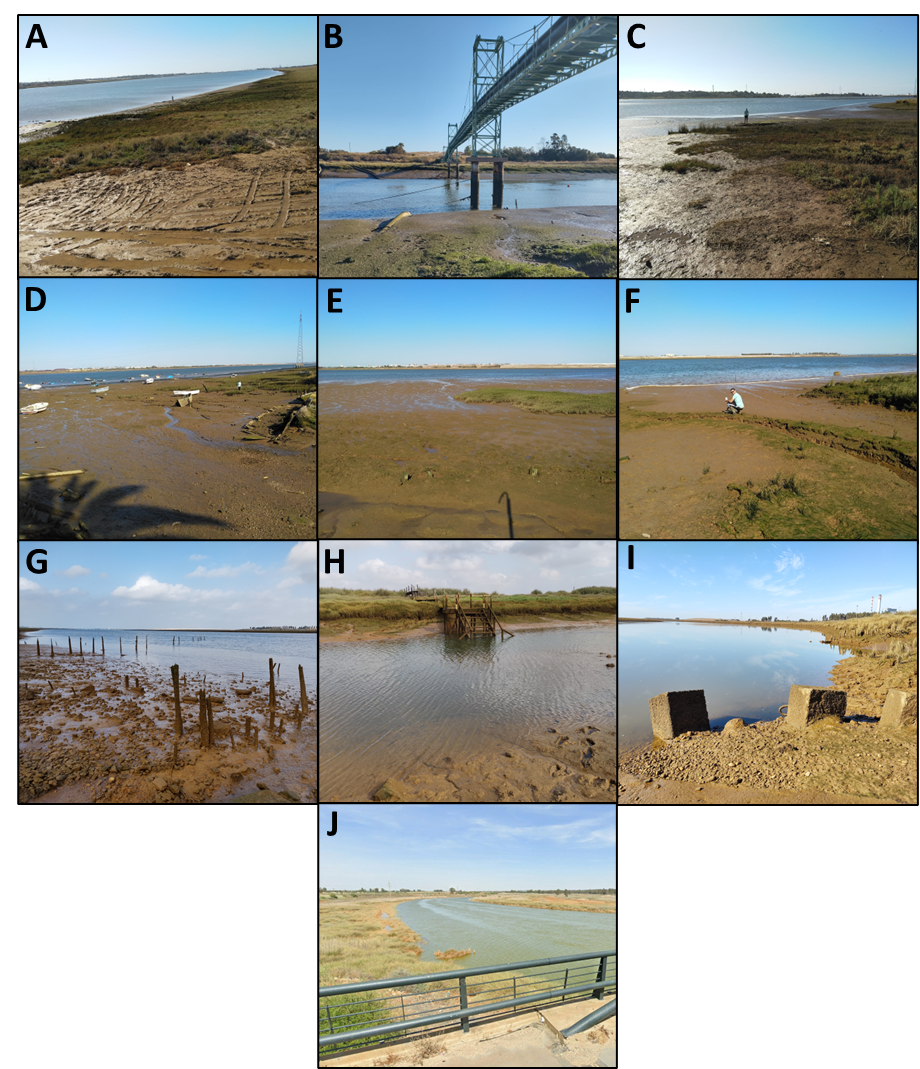


Fig. S1. Pictures of the sampling points. A: point 1; B: point 2; C: point 3; D: point 4; E: point 5; F: point 6; G: point 7; H: point 8; I: point 9; J: point 10.


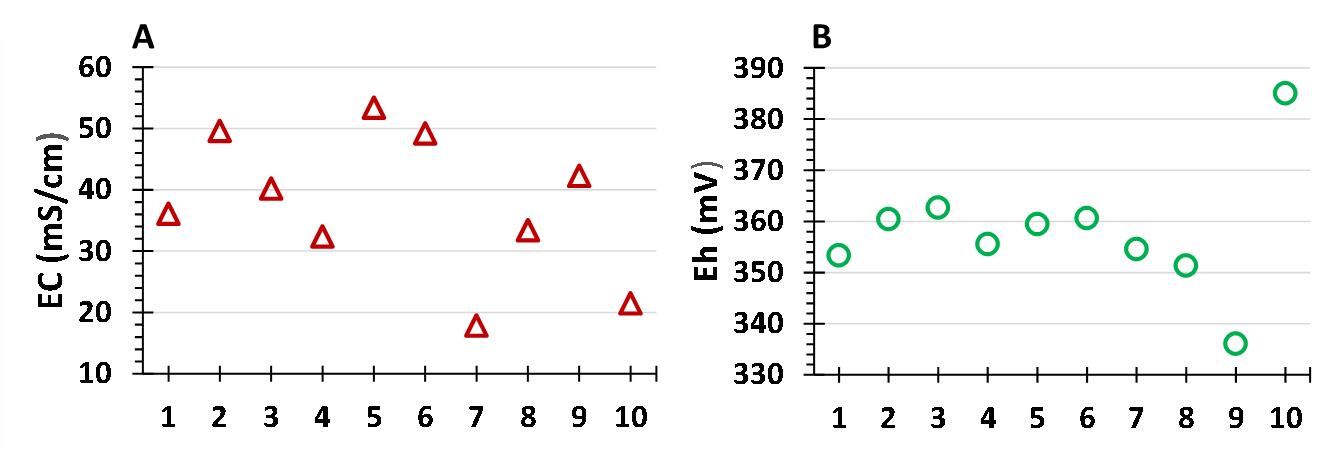


Fig. S2. Values of EC (A) and Eh (B) in the estuarine sediment samples.


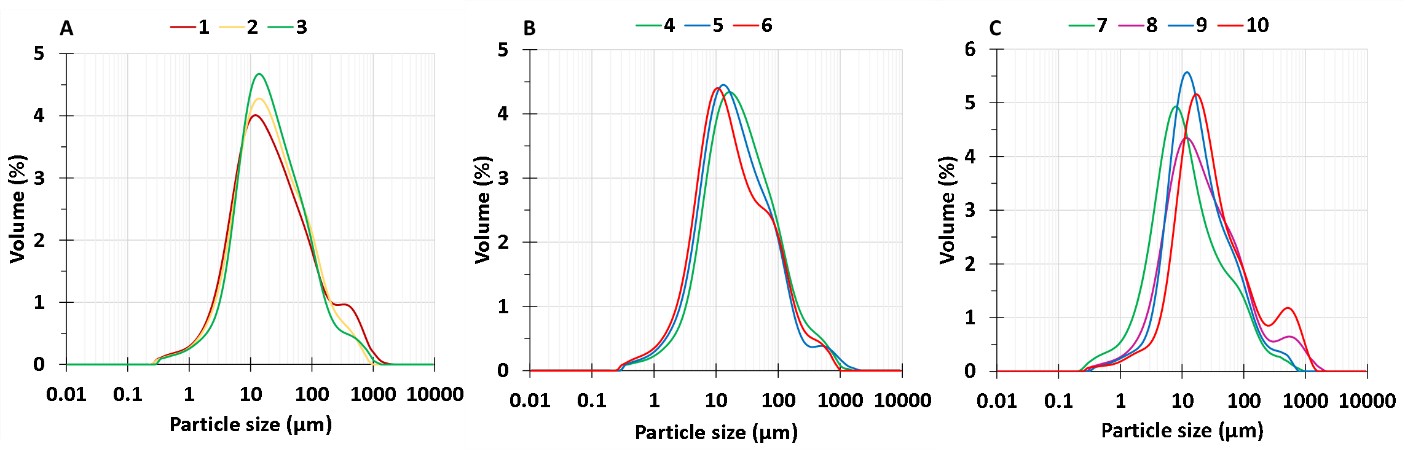


Fig. S3. Particle size distribution curves of the samples.


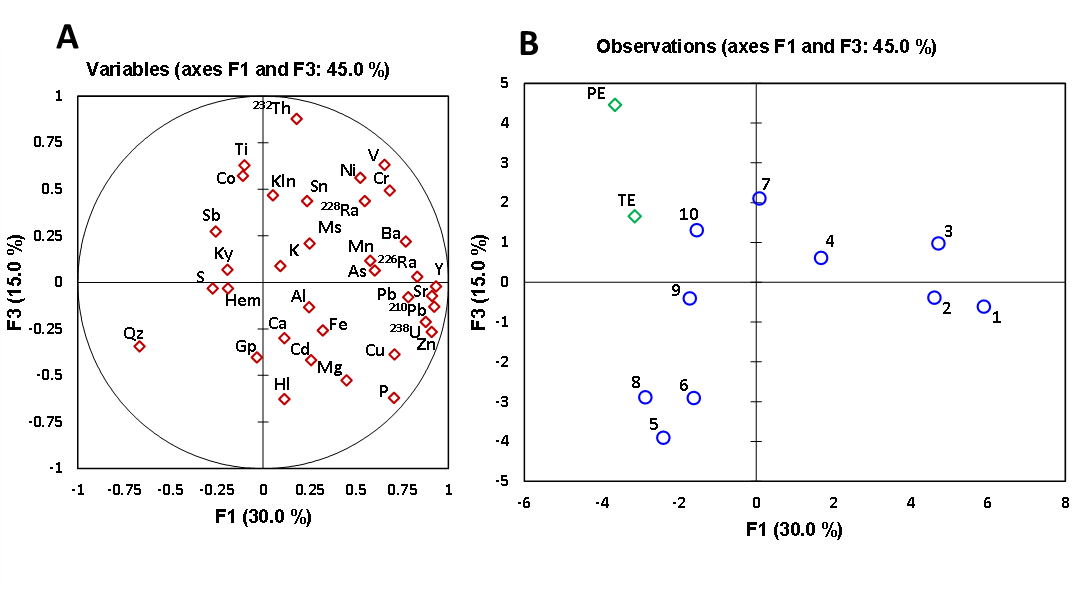


Fig. S4. PCA results of F1 vs F3. A: Loading plot of variables and B: score plot of observations. PE: Piedras estuary. TE: Tinto Estuary.


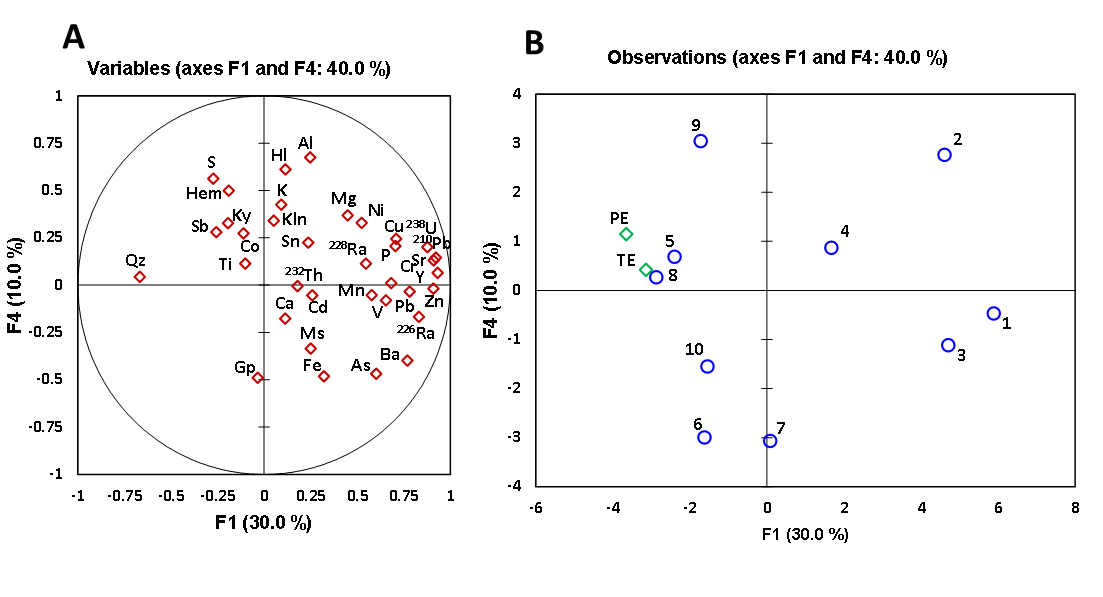


Fig. S5. PCA results of F1 vs F4. A: Loading plot of variables and B: score plot of observations. PE: Piedras estuary. TE: Tinto Estuary.
